# Convergences and Gaps between Environmental Ethics, Climate Ethics, and Research Ethics: A Scoping Review

**DOI:** 10.1007/s11948-025-00575-8

**Published:** 2026-01-12

**Authors:** Michel Bourban, Dominic Lenzi, Mads P. Sørensen, Rachel Fishberg, Jan Mehlich, Fabian Fischbach, José Luis Molina, Kasandra I. H. M. Poague, Alexandra Csábi, Rose Heffernan, Rosie Hastings, Anaïs Rességuier

**Affiliations:** 1 Philosophy Section, University of Twente, Enschede, The Netherlands; 2 The Danish Centre for Studies in Research and Research Policy, Department of Political Science, Aarhus University, Aarhus, Denmark; 3 Center for Life Ethics, University of Bonn, Bonn, Germany; 4 German Reference Centre for Ethics in the Life Sciences, University of Bonn, Bonn, Germany; 5 Department of Social and Cultural Anthropology, Autonomous University of Barcelona, Barcelona, Spain; 6 LISA (ICT, library and archiving services), University of Twente, Enschede, The Netherlands; 7 Center for Innovation Systems and Policy, Austrian Institute of Technology, Vienna, Austria; 8 Women Engage for a Common Future, Munich, Germany; 9 Department of Ethics, Law and Humanities, Amsterdam University Medical Center, Amsterdam, The Netherlands; 10 Trilateral Research Ltd, Waterford, Ireland

**Keywords:** Environmental justice, Environmental ethics, Climate justice, Research ethics, Innovation, Technology

## Abstract

Technological innovation is a double-edged sword for the environment. While it can significantly reduce environmental and societal risks of harm, technological progress is often a major contributor to environmental degradation. This suggests the need for an anticipatory approach to research oversight which can mitigate the harmful and contradictory effects of research and innovation. In this article, we conduct a scoping review to identify cross-cutting concepts between environmental ethics, environmental justice, climate ethics, climate justice, and research ethics and integrity, focusing on literature on research and technological innovation. This article identifies an important gap between research ethics and these other topic areas. It also demonstrates the importance of updating existing research ethics frameworks to incorporate environmental concerns evident in environmental ethics and climate justice. Finally, it reveals an over-representation of studies focused upon climate change at the expense of other environmental issues, and of research on the ethics and governance of geoengineering, which we suggest is not a generalisable model for governance of research.

**Supplementary Information:**

The online version contains supplementary material available at 10.1007/s11948-025-00575-8.

## Introduction

Technological innovation is a double-edged sword for the environment. It can significantly reduce environmental and societal risks of harm, such as when renewable energies replace fossil fuels. At the same time, technological progress is often a major contributor to environmental degradation. For instance, electric mobility can reduce greenhouse gas (GHG) emissions but requires mineral resources for batteries, the mining of which can create additional harms. Technologies can also enable progress in relation to one environmental objective but set back others, such as when artificial intelligence (AI) is put to work in the context of conserving endangered species, but doing so comes at the cost of a large carbon footprint. This double-edged nature suggests the need for an anticipatory approach to research oversight which can mitigate the harmful and potentially conflicting effects of research and innovation (R&I).

Because of the significant environmental and climate impact of R&I activities, certain research organisations and institutions have begun to require researchers to consider the environmental impact of their research, and ways to reduce it, as part of the research process and ethics approval. For example, the newest version of the Helsinki Declaration states that “Medical research should be designed and conducted in a manner that avoids or minimizes harm to the environment and strives for environmental sustainability” (WMA, 2024, p. 2). Likewise, the European Code of Conduct for Research Integrity (ALLEA, 2023) mentions respect for ecosystems and the environment as one of its fundamental principles. Research funding institutions have also recognised the need to consider environmental ethics dimensions in the evaluation and assessment of R&I activities. For example, in its guidance note for ethics self-assessments, the European Commission (EC) mentions that research activities must comply with the “precautionary principle and legislation on nature conservation and pollution control”, which implies proving “that a new technology will not harm the environment” (European Commission, 2021, p. 35).

Climate change is a paradigm case in which policies enacted now will either reduce or increase the risks of future warming. For this reason, the field of climate ethics has explored in detail the justice requirements of climate action, with a particular focus on future generations (e.g., Gardiner et al., 2010; Shue, 2014). Intergenerational justice has also long been central to how sustainability is understood. Climate ethics also highlights how technologically mediated risks may be imposed upon future generations, including the research and deployment of climate engineering techniques (e.g., Gardiner, 2010). Similarly, the interdisciplinary discourse about environmental justice highlights the unequal harms and risks of a wide range of policies and technologies spanning agriculture, industry, and conservation. Finally, environmental ethics has explored the moral status of non-human nature, including individual organisms (sentient and non-sentient), species, and ecosystems (for an overview, see Palmer et al., 2014). Because many R&I contexts impact both people and non-human nature, each of these discourses seems to offer highly relevant considerations for anticipatory governance of research.

Applied ethics in general has gained an increasingly important role in R&I processes in the past two decades (see Nyholm, 2023 for an overview). Integrating ethical expertise and knowledge into both technology development and policy has been the mission of various “transdisciplines”, such as technology assessment (Grunwald, 2024), science and technology studies (Felt & Irwin, 2024), and innovation research (De Vries, 2021). As distinct “epistemic regimes” of academic scholarship, environmental ethics and climate ethics both have the potential to serve as orientational knowledge in practice domains such as research, engineering, and technology development (Ott, 2021). The translation of principles and concepts into methods and tools (including ethics-by-design, value-sensitive design, responsible research and innovation, to name but a few) has made significant progress in recent years. In the same way that medical ethics can inform the development and design of service robots for hospitals by recognising patient autonomy through the inclusion of a visible off-button, environmental ethics and climate ethics insights might also enrich practically-oriented R&I discourses.

However, this presupposes an understanding of how concepts in research ethics and climate and environmental ethics relate to one another. We are not aware of any literature attempting to bring these academic discourses together, or to understand the areas of overlap that already exist between them. To make progress in the anticipatory governance of R&I activities that raise ethical concerns due to their environmental impacts, the present study conducts a systematic literature review of the key topic areas of research ethics and integrity, climate ethics and justice, environmental ethics, and environmental justice, in the context of R&I. Our objective is (1) to examine how these key topic areas are currently conceptualised in the literature, and (2) to explain how they relate to each other, highlighting major convergences and gaps between them.

Although research integrity is often the subject of separate guidance from that of research ethics, we treat the former as a subset of the latter because our search strings ultimately found insufficient literature to disaggregate them; for this reason, we use the general category of “research ethics and integrity”. The same applies to climate justice and climate ethics: although they can be discussed separately, in the context of R&I climate justice is discussed much more frequently than climate ethics; for this reason, we use the general category of “climate ethics and justice”. There was however enough literature on environmental justice and environmental ethics to keep these two categories separate.

The understanding of environmental justice used in this review was intentionally broad, with no restriction on research disciplines, in order to recognise the pluralism that exists concerning environmental values and worldviews (Pascual et al., 2023). We also recognise theoretical and conceptual pluralism in each of our topic areas and thus do not limit results to research in philosophy or ethics. We map similarities and gaps in this literature and in literature on research ethics and integrity, as well as concepts and issues in wider sustainability science literatures with implications for R&I. In this way, our review sought to answer the following research question:

*What are the cross-cutting concepts in environmental justice, environmental ethics, climate ethics and justice, and research ethics and integrity, in the context of research, technology and innovation?*

First, we explain the search methodology for identifying and analysing the relevant literature. The Results section presents the main findings of the literature review in relation to the main objective. After explaining how the key topic areas are conceptualised in the literature, this also investigates how these key topic areas are related to each other through a bibliometric mapping of the included studies and through an analysis of the cross-cutting concepts that emerged. The Discussion then reflects upon the main findings by providing a future research agenda and acknowledging the limitations of our scoping review. We also suggest that one of our most prominent examples, namely geoengineering, is unlikely to provide generalisable guidance for integrating environmental and climate ethics into research ethics.

## Systematic Review Method

We adopted a scoping review method because this type of literature review is well-adapted for mapping concepts and investigating how they relate to one another, as well as for identifying gaps between different research fields (Peters et al., 2015; Sutton et al., 2019). We followed best practices for scoping reviews based upon Arksey and O’Malley (2005). The review was performed according to the Preferred Reporting Items for Systematic Reviews and Meta-Analyses Extension for Scoping Reviews (PRISMA-ScR) protocol.

### Article Selection

We looked for concepts that are related to or that represent aspects of: environmental ethics, environmental justice, climate ethics and justice, and research ethics and integrity. While these topics could be reviewed more broadly, we only included results that considered them in relation to contexts of research, innovation and technology.

We used three databases to cover a wide range of the academic literature on the key topic areas: Scopus, Web of Science, and Philosopher’s Index. We included all literature that mentioned the concepts outlined. There was no restriction on the geographical location of studies or research disciplines. The review considered all publications without a time limitation. We included only peer-reviewed research articles, books, book chapters, and review articles.

We included literature written in English, French, German, and Spanish, although the search was performed solely in English. This means that French, German and Spanish literature with titles and abstracts available in English identified using English language search terms have been included and analysed. Because of the plurality and complexity of topics covered by this review, multiple iterations of the search strings were tested to find the formulation most adapted to our objectives. A separate search string was also formulated to capture the plurality of perspectives in the literature on environmental values, in order to include literature on indigenous and local knowledge. In doing so, we adapted an existing search string utilised by the IPBES in a review of indigenous and local values (Athayde et al., 2022), adding keywords relevant for this scoping review. Our searches were done in the three databases on 7^th^ May 2024 (the final search queries are in Table S1 in the Supplementary Materials; the intercultural search string was only used in Scopus). We discuss limitations related to these databases and search queries in the Discussion section. 

### Analytical Procedure

The review process took place in three stages: first, publications were screened based on title and abstract against inclusion and exclusion criteria; second, the remaining subset of publications was screened based on the full text against elaborated inclusion and exclusion criteria (see Table S2 in the Supplementary Materials); third, the data relevant for the review was extracted based on a data extraction template.

All authors took part in the three steps of the review process. At stage 1, a sample of publications was initially distributed for a pilot study to validate the inclusion and exclusion criteria. The initial inclusion and exclusion criteria were modified inductively in light of the pilot study and were agreed upon before the start of stage 1. The same process took place at stage 2. To reduce bias, two reviewers were randomly allocated to each publication for each step, and any disagreements regarding the inclusion or exclusion of a publication were subsequently discussed to reach consensus (the number of publications included and excluded at each stage is displayed in figure S1 in the Supplementary Materials). Various attributes of each publication included for the review were recorded in data extraction files during stage 3. Frequencies, co-occurrence of study characteristics and data visualization were performed with Excel, VOSviewer and Tableau version 2024.3.0.

## Results

### Conceptualisations of key topic areas

A total of 855 publications were screened in the first step of the review. In the second step, 339 studies were screened, and a total of 156 studies were selected for the third and final step (see Table S3 in the Supplementary Materials for a list of all the included studies). We were as inclusive as possible: as long as a publication provided an explicit definition of the key topic area(s) discussed, or as long as it used the key topic area(s) as part of the methods/approaches or as part of findings/conclusions, it was included.^1^  

Explicit definitions of the key topic areas were found in 43% of the reviewed literature; as such, a majority lacked clear definitions of these topic areas (57%), even though they played a central role in the study. Among articles featuring definitions of key topics, the review found limited evidence of shared understandings in the literature surveyed, with considerable variation in basic definitions, authoritative citations, or applications to areas of R&I.

The key topic area that was the most discussed was **environmental justice**, with 33% of the literature mentioning it, and 29% providing an explicit definition (see Table 1). Despite this, relatively few shared definitions or understandings across the literature were found. A small group of studies (3%) refer to the definition of environmental justice provided by the US Environmental Protection Agency (EPA, 1998). This definition emphasises distributive justice – in terms of allocation of negative environmental consequences – and participatory or procedural justice – in terms of the inclusion of all people in the political process leading to environmental decision making. A larger group of studies (10%) use the definition of environmental justice provided by Schlosberg (2002, 2004, 2007, 2013). This also highlights distributive and procedural justice but adds recognition of the diversity of concerns and pluralistic values in environmental justice, and an interest in capabilities conceived as the basic needs and functionings of individual and collective agents. Aside from these two sources, few common definitions of environmental justice were identified.

**Table 1:** Key conceptual components of environmental justice from the included literature

Conceptual element	Definition	Mentions	References
Distributive justice	Involves the fair distribution of environmental benefits and burdens across individual and collective agents. It ensures both that individuals and groups have access to a fair share of environmental benefits and that no individual or group is exposed to an inequitable allocation of negative environmental impacts.	7	(Batel & Devine-Wright, 2017; Behrsin, 2020; Bettini et al., 2020; Bush & Doyon, 2023; Kurochkin et al., 2021; McCauley & Heffron, 2018; Ziegler et al., 2023)
Procedural justice	Emphasizes the importance of inclusive, transparent, and democratic decision-making processes. It ensures that all the stakeholders affected by environmental policies have meaningful participation opportunities in the political process guiding the design and implementation of these policies. Particular attention is paid to those who have historically been marginalized or excluded from the decision-making process.	7	(Batel & Devine-Wright, 2017; Behrsin, 2020; Bettini et al., 2020; Bush & Doyon, 2023; Kurochkin et al., 2021; McCauley & Heffron, 2018; Ziegler et al., 2023)
Recognition justice	Focuses on acknowledging and valuing the diverse perspectives and experiences of different social, cultural, ethnic, racial, and gender groups, and addressing any forms of misrecognition or non-recognition. It is related to procedural justice, with the idea that all groups with a stake in environmental decision-making processes should have their interests and positions recognized and represented.	5	(Batel & Devine-Wright, 2017; Behrsin, 2020; Bettini et al., 2020; Bush & Doyon, 2023; Ziegler et al., 2023)
Restorative justice	Involves addressing past environmental harms through compensation, restitution, and resolution for people and communities affected by such harms, as well as the remediation of damages.	5	(Kaul et al., 2023; Kurochkin et al., 2021; McCauley & Heffron, 2018; Tomblin, 2009; Vaishnav, 2023)
Justice as capability	Ensures that individuals and communities do not only have the necessary resources, but also the necessary opportunities to convert these resources to live healthy, safe, flourishing, and dignified lives. Promoting capabilities fulfillment includes providing access to environmental benefits but also protecting from environmental harms.	2	(Behrsin, 2020; Bush & Doyon, 2023)

Next, a total of 27% of the studies mentioned **environmental ethics**, with 22% providing explicit definitions of this as a field of study, or explicit definitions of specific concepts within environmental ethics. Since the question of ethical considerability of non-humans plays a crucial role in environmental ethics, the topic of non-anthropocentrism, i.e. the rejection of human-centredness, was prominent in the literature surveyed (11%). Anthropocentrism and the different forms of non-anthropocentrism are all defined in Table 2 below. A somewhat disconnected literature on the concept of corporate environmental ethics was also identified, with eight studies utilising this approach to develop their argument (5%). This was defined most often by reference to a review article on environmental ethics written by Palmer et al. (2014), which does not mention the topic of corporate environmental ethics. This suggests that the concept lacks a clearly discernible meaning.

**Table 2:** Key conceptual components of environmental ethics from the included literature

Conceptual element	Definition	Mentions	References
Ecocentrism	Collective beings, such as ecosystems and species, have intrinsic value.	10	(Baum & Owe, 2023; Bhardwaj, 2006; Biasetti & Grigoletto, 2023; Green, 2024; King, 2006; Okada & Watanabe, 2008; Petit & Guillaume, 2018; Shrader-Frechette, 2005; van Wynsberghe & Donhauser, 2018; Verharen et al., 2021)
Biocentrism	All living beings, including plants, have intrinsic value.	8	(Baum & Owe, 2023; Bhardwaj, 2006; Biasetti & Grigoletto, 2023; Green, 2024; King, 2006; Okada & Watanabe, 2008; Shrader-Frechette, 2005; van Wynsberghe & Donhauser, 2018)
Anthropocentrism	Non-human natural beings only have instrumental value, in the sense that their value derives from their usefulness to humans; only human beings have intrinsic value and should be considered as ends in themselves.	6	(Bhardwaj, 2006; Cera, 2020; Green, 2024; Petit & Guillaume, 2018; Shrader-Frechette, 2005; van Wynsberghe & Donhauser, 2018)
Ecofeminism	Focuses on the gendered nature of environmental inequalities and on the relation between people and places, recognizing how power and culture are infused in place-based networks.	6	(Lloro-Bidart & Finewood, 2018; Mahoney, 2016; Roe & Zavar, 2021; Shrader-Frechette, 2005; Stephens, 2024; Wiseman, 2022)
Sentientism	All sentient non-human animals have intrinsic value; sentient beings capable of experiencing pleasure (interest satisfaction) and pain (setback in interest) have moral standing.	4	(Baum & Owe, 2023; King, 2006; Okada & Watanabe, 2008; Shrader-Frechette, 2005)
Environmental virtue ethics	Focuses on characters and dispositions we ought to adopt regarding the environment, complementing the ethic of action with an ethic of character, and asking questions related to the good life rather than the right action.	4	(Anthony, 2012; Coeckelbergh, 2011; Dzwonkowska, 2017; Mahoney, 2016)
Deep ecology	The natural environment as a whole, not only species or ecosystems, has intrinsic value. Deep ecology themes include showing respect for nature, being one with nature, and engaging in environmental activism.	2	(Okada & Watanabe, 2008; Shrader-Frechette, 2005)

Turning to **climate justice,** and the closely related concept of **climate ethics,** we found even less evidence of shared understandings across the literature reviewed (see Table 3). A total of 21% of the studies reviewed discussed climate justice or climate ethics, with 13% providing definitions of these terms. Contrary to our expectations, the work of Shue (1993, 2005, 2014) and Caney (2009, 2010, 2014), which is particularly influential in climate justice and responsibilities for mitigation, was cited in merely four articles in the literature surveyed. Three articles cited the work of Adger and colleagues on adaptation to climate change (Adger et al., 2006; Paavola & Adger, 2002). Instead, most studies surveyed offered their own definitions of climate justice, supported by different references in many cases (and sometimes by none at all). More specific concepts within climate justice research were also found, such as the polluter pays principle and the ability to pay principle. Other literature emphasised transformative change, gender justice, or power relations as central to climate justice.

**Table 3:** Key conceptual components of climate justice from the included literature

Conceptual element	Definition	Mentions	Reference
Burden-sharing justice	Refers to the allocation of responsibility for addressing climate change between countries and across generations, with the objective to obtain a fair distribution of the costs of climate policies such as mitigation, adaptation, and loss and damage policies.	9	(Bourban, 2022; Bush & Doyon, 2023; Hilser et al., 2024; Khayat, 2023; McAllister et al., 2014; McCauley & Heffron, 2018; Okereke, 2010; Popke et al., 2016; Ying & Sovacool, 2021)
Procedural justice	Refers to fair participation in decision-making processes that shape climate policies.	5	(Forsyth & McDermott, 2022; Hilser et al., 2024; McCauley & Heffron, 2018; Okereke, 2010; Popke et al., 2016)
Restorative justice	Aims at redressing the past harms of extractive activities, coloniality, economic injustice, and systems of exploitation that maintain and increase the vulnerability to climate impact of people and communities suffering from historic injustices.	3	(Kinol et al., 2023; Okereke, 2010; Stephens & Sokol, 2023)
Recognition justice	Refers to the acknowledgement of people’s diverse needs and experiences in the design and implementation of climate policies.	2	(Hilser et al., 2024; Popke et al., 2016)
Harm avoidance justice	Aims at ensuring that the most vulnerable to climate impacts are protected and that climate policies do not result in further harming the global poor.	1	(Bourban, 2022)

Finally, we reviewed publications on **research ethics and integrity**, but only insofar as these included an explicit and clear consideration of environmental ethics/justice or climate ethics/justice. We anticipated that only a limited number of studies on research ethics and integrity would be found, but we were surprised by how little of this key topic area was finally included. Only 6% of the literature included had explicit definitions pertaining to research ethics and integrity, and these came exclusively from the perspective of **responsible research and innovation** (RRI). RRI's major aim is to align the governance of novel fields of science and technology with societal values by creating, maintaining, and developing the dialogue between scientific production, technological innovation, and society, for instance through deliberative conversations between academic and non-academic stakeholders. RRI also reflects the idea that research and innovation should also contribute to sustainability outcomes. For instance, Biddle (2017) cites the European Commission’s definition of RRI, which states: “Responsible research and innovation is an approach that anticipates and assesses potential implications and societal expectations with regard to research and innovation, with the aim to foster the design of inclusive and sustainable research and innovation.”

**Fig. 1:**
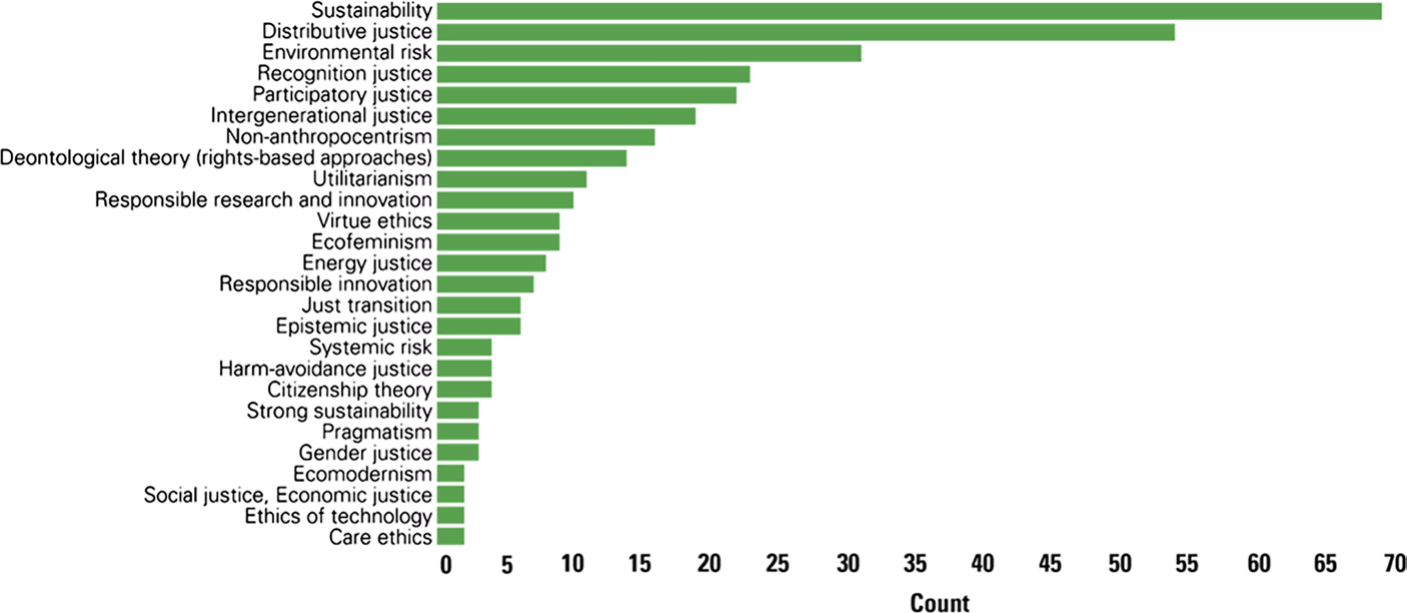
Specific ethical or theoretical concepts applied to key topic areas

Figure 1 provides an overview of the most discussed ethical and theoretical concepts (i.e. those mentioned at least twice).

To appear in these results, these concepts did not need to have been clearly defined, nor even to play an important role in the analysis. Instead, it was sufficient for these concepts to be mentioned in the article reviewed. An analysis that looked more closely at how some of these notions were defined and what role they played in linking the key topic areas is provided in the following sub-sections.

The concept that is the most often mentioned – in 47% of the studies – is sustainability. Given the scope of our review this is unsurprising, although it is still relevant to note how often it appears as this indicates that in the R&I context there remains a strong connection between sustainability and ethics and justice-related issues related to environmental problems.

Distributive justice (mentioned in 44% of the literature), recognition justice (15%), and participatory justice (14%) also played a major role. This supports the findings above regarding the key conceptual elements of environmental justice and climate justice in the literature surveyed. Environmental risk (20%) and intergenerational justice (12%) were also important ethical and theoretical concepts. As explained below, together with distributive justice, recognition justice, and participatory justice, intergenerational justice represents a concept that cuts across two or more key topic areas.

Non-anthropocentrism (10%), virtue ethics (6%), and ecofeminism (6%) played a somewhat important role in the conceptualisation of environmental ethics. Responsible research and innovation (6%) and responsible innovation (4%) were also mentioned in the conceptualisation of research ethics and integrity, although even more sparingly.

**Fig. 2:**
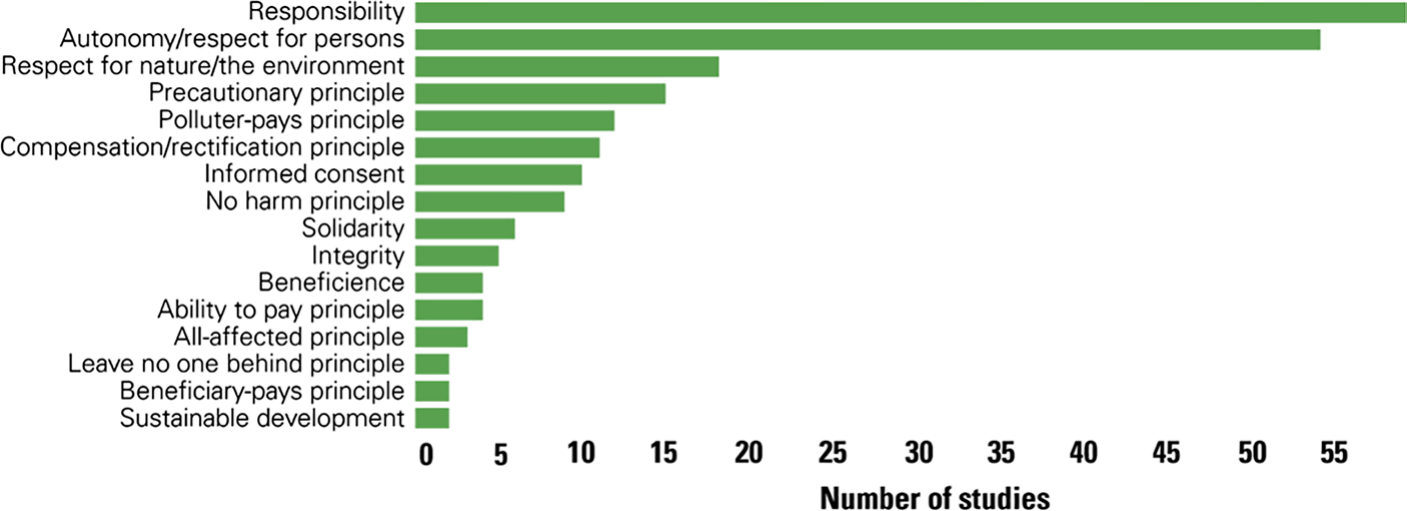
Principles or values mentioned in studies

Figure 2 provides an overview of the most discussed principles and values (i.e. those mentioned at least twice).

The two most influential values found in the selected literature were responsibility (37.8%) and autonomy (or respect for persons) (34.6%). This may be because responsibility and autonomy have many different meanings and can be applied in many different contexts. Responsibility, a major cross-cutting concept discussed below, was linked to the polluter-pays principle (7.7%), the ability to pay principle (2.6%), and the beneficiary pays principle (1.3%) in the context of allocating responsibility to different individual and collective agents, especially nation-states.

Autonomy is often mentioned in the bioethics literature included in this review. Bhardwaj (2006, p. 170) conceives autonomy as the “guiding principle for recognition of human capacity for self-determination and independency in decision-making”. It is mentioned alongside other bioethics principles such as beneficence (the intention to do good and the practice of good deeds), non-maleficence (the obligation not to inflict harm), and justice (fair treatment and equity). There is some overlap with the literature on environmental justice, as non-maleficence is based on the no harm principle (5.8%), which could be extended to environmental harms (Vaishnav, 2023), and justice as fair treatment is very close to procedural or participatory justice.

Two other influential principles were the precautionary principle (9.6%), and respect for nature or the environment (11.5%), which is often conceived as an attitude or character trait necessary to live an environmentally virtuous life.

### Relations between key topic areas

**Fig. 3:**
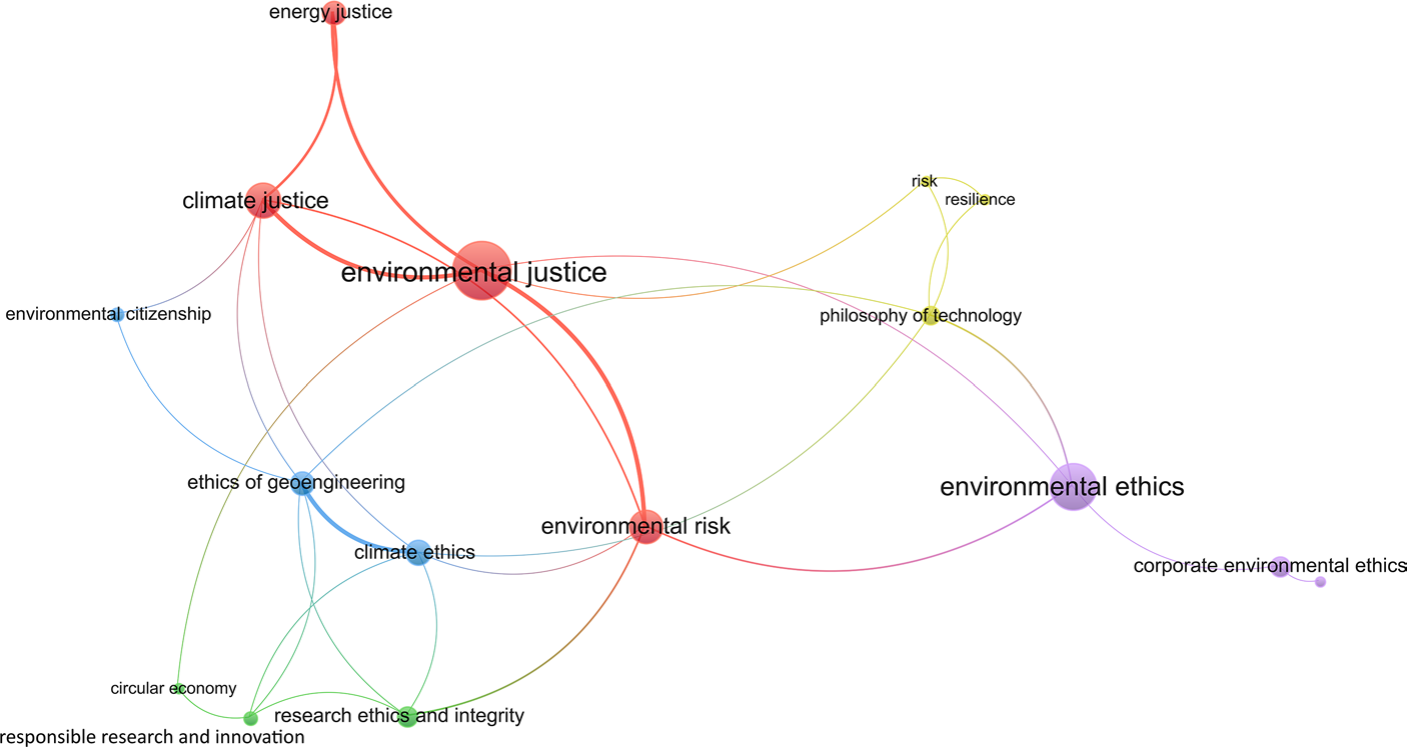
Bibliometric mapping of the studies reviewed

This sub-section investigates how the key topic areas conceptualised in the previous section relate with each other. A bibliometric mapping of the reviewed studies is shown in Fig. 3, based on the most frequently mentioned terms, using the VOS Viewer software. Only terms that were mentioned at least twice appear.

The five main clusters based on keyword occurrences are environmental justice (red), environmental ethics (purple), climate ethics (blue), research ethics and integrity (green), and philosophy of technology (yellow). These clusters were created from the default key topic areas reviewers could choose from in the data extraction template, as well as other topics reviewers added, such as philosophy of technology.

The larger the node, the higher the number of occurrences of the concepts in the reviewed literature (e.g., 54 occurrences for environmental justice, 35 for environmental ethics, and 20 for climate justice). The concepts that are directly connected in the same cluster co-occurred in a least two papers (e.g., ethics of geoengineering with climate ethics); this also applies to concepts that co-occurred in different clusters (e.g., ethics of geoengineering and RRI). The stronger the link between two concepts, the thicker the line that is used to display the link. Environmental justice, climate justice, ethics of geoengineering, and climate ethics have a total of 6 links each, showing that they are highly interconnected concepts; environmental risk and philosophy of technology have a total of 5 links, and research ethics and integrity and RRI have 4 links, showing they are more moderately interconnected; the other concepts played a smaller role in connecting the key topic areas between themselves.

The resulting network shows that environmental justice and climate justice are connected directly, but also indirectly through energy justice. Interestingly, environmental justice and environmental ethics are not directly connected to research ethics and integrity, but indirectly through the notion of “environmental risk.” Environmental justice is however directly connected to the notion of “circular economy,” which is part of the research ethics and integrity cluster. Climate ethics and the ethics of geoengineering are both directly connected to research ethics and integrity and RRI, while the ethics of geoengineering also connects climate justice with research ethics and integrity and RRI. This shows that environmental risk, the ethics of geoengineering, and circular economy play an important role in connecting different key topic areas. Philosophy of technology connects climate ethics and the ethics of geoengineering to environmental ethics. Environmental justice and philosophy of technology are however indirectly connected through the notions of “risk” and “resilience”. Corporate environmental ethics appears as separate from the rest of the network, which illustrates its lack of connection with the topics and concepts discussed in this review.

While Figure 3 does not directly provide cross-cutting concepts it shows how often these topic areas are mentioned and how they are interconnected. To identify cross-cutting concepts, it was necessary to examine in more detail the data extracted from the studies, and sometimes to go back to the studies themselves to get more specific information.

### Cross-cutting concepts

Finding cross-cutting concepts in the key topic areas was one of the main objectives of our review. Here we define and discuss the main cross-cutting concepts identified. We also provide illustrations of how they are used in the literature. For a concept to be cross-cutting, we required it to meet the following criteria:

- *Relevance across key topic areas:* the concept appears and is used in the analysis in more than one topic area;
- *Capacity to bridge different knowledge domains:* the concept helps integrate the issues and topics discussed in different research fields;
- *Transferability:* the concept can be adapted and used to frame research questions in different research fields, even if it is not defined in the same way.

As Table 4 shows, there are more cross-cutting concepts between environmental justice and climate justice than between environmental ethics/justice, climate ethics/justice, and research ethics and integrity. The most mentioned cross-cutting topics in the more restricted key topic areas (i.e. environmental justice and climate justice) were distributive, procedural and recognition justice. However, in the expanded key topic areas (i.e. environmental ethics/justice, climate ethics/justice and research ethics and integrity) the cross-cutting topics most often mentioned were sustainability and responsibility.

**Table 4:** Cross-cutting concepts, based on their mentions

Cross-cutting concept	Key topic areas	Mentions
Distributive justice	Environmental justice and climate justice	54
Recognition justice	Environmental justice and climate justice	23
Procedural justice	Environmental justice and climate justice	22
Intergenerational justice	Environmental justice and climate justice	19
Polluter-pays principle	Environmental justice and climate justice	12
Indigenous perspectives or traditions	Environmental justice and climate justice	10
Energy justice	Environmental justice and climate justice	10
Restorative justice	Environmental justice and climate justice	8
Feminism	Environmental justice and climate justice	6
Sustainability	Environmental ethics/justice, climate ethics/justice and research ethics and integrity	69
Responsibility	Environmental ethics/justice, climate ethics/justice and research ethics and integrity	59
Precautionary principle	Environmental ethics/justice, climate ethics/justice and research ethics and integrity	15
Geoengineering	Environmental ethics/justice, climate ethics/justice and research ethics and integrity	14
Citizenship	Environmental ethics/justice, climate ethics/justice and research ethics and integrity	8
Epistemic justice	Environmental ethics/justice, climate ethics/justice and research ethics and integrity	4

#### Environmental justice and climate justice

As clearly illustrated by Tables 1 and 3 above, the following concepts cut across environmental justice and climate justice:

- **Distributive justice**, which focuses on the fair distribution of environmental and climate burdens, ensuring that marginalized people and communities do not bear disproportionate environmental and climate risks and harms.
- **Procedural justice**, which emphasizes the importance of inclusive and fair decision-making processes, ensuring that all stakeholders, especially marginalized groups, have a voice in environmental and climate-related decisions.
- **Recognition justice**, which acknowledges the need to recognize and respect the identities, experiences, and rights of marginalized communities, including Indigenous peoples, in environmental and climate justice frameworks.
- **Restorative justice**, which addresses historic injustices caused by past environmental and climate endangering economic activities, and aims to redress harms and damages caused by these activities and their long-lasting effects.

A fifth cross-cutting concept is **intergenerational justice**. This is not surprising, since we noted at the outset that intergenerational justice has long been a core feature of sustainability. Much of the discussion in environmental justice and climate justice is about the specifications of the responsibilities we have towards future generations, typically in terms of distributive justice, with the idea that environmental benefits and burdens (especially environmental risks and hazards, but also the risks raised by environmental and climate technologies) should be distributed fairly across generations.

A sixth cross-cutting concept is the **polluter-pays principle**. The idea that individual, but mostly collective agents should pay for the consequences of their polluting activities is very common in the environmental justice and climate justice literature. The polluter-pays principle is a principle of distributive justice supporting that emitters must pay because of their past and present contribution to climate change (Ciplet & Roberts, 2017). As Popke et al. (2016, p. 71) stress, the polluter-pays principle has a strong backward-looking component to attribute responsibilities to address climate change, since some agents have made substantial contributions to climate change while others have contributed much less.

A seventh cross-cutting concept in environmental and climate justice literatures was **indigenous perspectives or traditions**. The studies reviewed did not cite standard definitions of what made something an indigenous perspective, but had in common an emphasis on the embeddedness of nature and culture in the local territory, denying the divide between humanity and nature, or humans and non-humans. Nonetheless, the decolonial perspective of the philosopher Kyle Powys Whyte (2017, 2018) informed four of these studies.

An eighth topic that links environmental justice with climate justice is **energy justice**. The most common understanding of energy justice came from Sovacool and Dworkin (2014), although this was merely cited in 4 out of 10 studies. This defines an energy-just world as a world “that equitably shares both the benefits and burdens involved in the production and consumption of energy services, as well as one that is fair in how it treats people and communities in energy decision-making” (Sovacool & Dworkin, 2014, cited in Kim et al., 2022, p. 24). Other understandings of energy justice came from Heffron and McCauley (Heffron, 2022; Heffron & McCauley, 2017; McCauley & Heffron, 2018) and Jenkins et al. (2016), which were cited in two studies. Interestingly, energy justice was more commonly understood in terms that were almost identical to Schlosberg’s view of environmental justice (Lloro-Bidart & Finewood, 2018; Roe & Zavar, 2021; Stephens, 2024).

The last cross-cutting concept was **feminism**. This highlights the gendered nature of both environmental and climate injustices and calls for gender diversity and equality in environmental and climate decision-making. A major feminist approach is the one of ecofeminism, which explains that social inequalities in terms of sexism, racism, and classism, and environmental inequalities in terms of air, land, and water pollution are often closely intertwined (Lloro-Bidart & Finewood, 2018). Three out of six studies cited ecofeminist theorist Val Plumwood (1988, 2003, 2008), who highlights how forms of oppression must be analysed alongside one another, as they are often mutually reinforcing and compounding. In this context, intersectionality is well suited to ecofeminist theory, defined as a “theory and approach of studying the relationships amongst numerous dimensions of social relationships, subject formations, and categories of power” (Hovorka, 2015, cited in Lloro-Bidart & Finewood, 2018).

#### Environmental ethics/justice, climate ethics/justice, and research ethics and integrity

We found a lower number of concepts that cut across all key topic areas. **Sustainability** appears as the most important one. As noted already, this is not surprising given the key role this concept has played in shaping political, social, economic, and ethical discussions on the environment. In contrast with the other cross-cutting concepts discussed already, it was difficult to find a common understanding of sustainability in the reviewed literature. This is also unsurprising as the definition of sustainability has been a source of disagreement for a long time. One particularly influential definition of sustainability is proposed by the Brundtland report, which defines sustainable development as “development that meets the needs of the present without compromising the ability of future generations to meet their own needs” (WCED, 1987 p. 8; cited in Briggle, 2009). From this starting point, different and often competing understandings of sustainability have emerged. For instance, some have emphasised the environmental dimensions of sustainability and called for radical changes in human attitudes towards nonhuman nature (e.g., Bassey, 2020). Others have emphasised the economic dimensions of sustainability with a focus on green human capital, green innovation, and green corporate environmental ethics (e.g., Chen & Chang, 2013). These two examples illustrate how complex and multifaced the concept of sustainability is, and how it can have different meanings depending on which topic it is applied to, or from which disciplinary perspective it is investigated.

The second most important cross-cutting concept was **responsibility**. As noted already, this concept plays a central role in environmental justice and climate justice, where the allocation of responsibility to address climate change and other environmental issues is a major research focus, and connects with discussions of the polluter pays principle, the ability to pay principle, and the beneficiary pays principle. Responsibility is also often used in the context of research ethics, where RRI plays an important role. Purvis et al. (2023) discuss the framework for RRI adopted by the EU in 2014 for the Horizon 2020 research programme, which highlights that the inclusion of women in research activities and research oversight processes is necessary but not sufficient for research to be conducted responsibly. For this reason, they recommend focusing on the AIRR framework as the main guiding principles for R&I activities in pursuit of a just transition to a circular economy, based on the core values of anticipation, inclusion, reflexibility, and responsiveness. This framework also connects responsibility in research ethics with environmental justice.

A third cross-cutting topic was the **precautionary principle**. Mentions of the precautionary principle were most common in environmental ethics with 7 mentions, while there were 3 mentions of it in relation to environmental justice, 2 mentions in relation to research ethics and integrity, and 2 mentions in relation to climate justice. The precautionary principle also featured in studies on RRI and environmental risk. There was no unified definition of this principle, but it is related to the notions of (environmental) risk and (environmental) harm, and the question of how to deal with uncertainties raised by technological innovation. The concept of “systemic risk” was sometimes used to refer to risks that endanger the functioning of vital systems such as infrastructure, supply chains, and healthcare systems (Hofbauer, 2023; Yearley, 2009). Alario and Freudenburg (2010) also introduced the concept of “Titanic risk” to complement the understanding of “risk society” (Beck, 1992; cited in; Alario & Freudenburg, 2010) to highlight that technological risks, such as toxic waste, nuclear technologies, and climate impacts, fundamentally reflect problems of inequity and inequality, in line with environmental justice concerns.^2^

A fourth cross-cutting concept was **geoengineering**, which covers both solar radiation management (SRM) and carbon dioxide removal (CDR). Geoengineering is defined as “deliberate large-scale manipulation of the planetary environment to counteract anthropogenic climate change” (Royal Society, 2009, cited in Muraca & Neuber, 2018; Pamplany et al., 2020). The conceptualisation of this object of research is itself a topic of debate, with some authors using “geoengineering” (e.g., Pamplany et al., 2020), others using “climate engineering” (e.g., Brooks et al., 2022) and others using alternative categorizations such as “land-based mitigation technologies (LMTs)” (Karki et al., 2023) or “negative emissions technologies (NETs)” (Mintz-Woo, 2023). The ethics of geoengineering, a sub-field of climate ethics and justice, focuses primarily upon the ethical and governance challenges of research into geoengineering techniques. It is clearly linked to research ethics, with two contrasting approaches on the permissibility and governance of research and deployment into geoengineering in the literature reviewed by Pamplany et al. (2020). The precautionary principle has been invoked by different authors to raise concerns over outdoor experimentation of some geoengineering techniques, with some calling for a moratorium on research.

While earlier literature on the ethics of geoengineering tended to focus on SRM, an emerging literature focuses on CDR (used mostly interchangeably with NETs) and/or LMTs. In a perspective relevant for research ethics, Nawaz and Satterfield (2024) provide a set of justice-related criteria for CDR projects. Karki et al. (2023) review ethical barriers to LMTs and discuss trade-offs in terms of land availability and competition with other land uses, noting risks of increasing food prices and food insecurity, along with risks of land grabbing, inequitable sharing of benefits and negative effects on vulnerable groups lacking access or secure land rights, including women, minority groups, and Indigenous peoples. Finally, 3 studies mentioned ethical issues reviewed by Lenzi (2018), such as the risk of CDR obstructing climate mitigation.

A fifth cross-cutting concept was **citizenship**. A first relevant application of this notion is the one of citizen science, a strategy for environmental protection that allows for the incorporation of environmental justice concerns from populations exposed to environmental hazards (Perovich et al., 2018; Scott & Barnett, 2009; Shulman et al., 2005; Wyeth, 2023). Citizen science engages the public in collecting scientific data, asking research questions, and finding patterns, for instance by monitoring air quality. This allows the incorporation of societal concerns into future assessment and regulation of technological innovation, for instance through hybrid knowledge production combining scientific knowledge and knowledge from lay communities, with lay knowledge being conceived as local, nonscientific, hard earned, less formally organized, and related to self-identity (Scott & Barnett, 2009). A second relevant application of the notion of citizenship is environmental or ecological citizenship (Bourban, 2022; Karlsson, 2012; Symons & Karlsson, 2018; Wong & Sharp, 2009). Environmental citizenship aims to reconcile environmental policy objectives with democratic commitments by prompting individuals to voluntarily choose more sustainable lifestyles and support collective efforts to protect the environment. Environmental citizenship is also related to the focus in climate ethics on individuals’ duties to reduce their carbon footprint and to promote collective action.

The sixth cross-cutting concept was **epistemic justice**, with one mention in research ethics and integrity (Arancio, 2023) and three mentions in papers discussing both climate ethics/justice and environmental ethics/justice (Arias Schreiber et al., 2022; Bush & Doyon, 2023; Mabon & Shackley, 2015). Epistemic justice supports the protection and incorporation of Indigenous knowledge, languages, stories, and songs into environmental policies. Epistemic justice calls for “alternative forms of being and seeing the world to be recognised as valid and valuable knowledge and includes the even greater challenge of translating this into real policy and practice and social justice” (Temper, 2019, p. 106, cited in Bush & Doyon, 2023). Interestingly, epistemic justice is framed in this literature as a complement to distributive, procedural, and recognition justice, which highlights the need to go beyond traditional approaches to environmental and climate justice to link this topic area with research ethics and integrity (e.g., Mabon & Shackley, 2015).

Epistemic justice was mentioned specifically in the context of using digital technologies for biodiversity conservation or climate policy. Three studies emphasised that the use of technologies collecting data needed to respect the rights of local and Indigenous peoples, including their rights to privacy, autonomy, (data) sovereignty, and (intellectual) property (Bettini et al., 2020; Cifuentes, 2023; Parris-Piper et al., 2023). This included the use of smart earth technologies monitoring and tracking environmental degradation, such as forest monitoring programs, drones, and satellite images. These studies also emphasised the importance of including local and Indigenous people in the decision-making process on how to implement these technologies. In this context, Bettini et al. (2020) proposed the concept of “digital justice”, which like energy justice was also defined in terms of distributive justice, participatory justice, and recognition justice.

#### Categorization of cross-cutting concepts

To conclude this section, we categorise the different cross-cutting concepts that have been identified. We present these categories from the most to the least abstract. Sustainability and indigenous perspectives or traditions represent **socio-ecological paradigms**: they link ecological and social systems, based on normative claims on how to live in these systems, thereby prescribing sustainable lifestyles and ways of living together. Feminism, the ethics of geoengineering, and citizenship theory represent **moral and political research fields**: they engage in critical reflection on existing institutions, focusing on how governance and power are exercised, and how existing policies and technologies should be changed. Distributive, procedural, recognition, restorative, and epistemic justice are **kinds of justice**: they represent different but complementary ways to think about the demands of justice. Finally, the polluter-pays and the precautionary principle are **principles of justice**: they represent normative rules that specify what is fair, equitable, or morally right. They qualify the demands of justice provided by the different kinds of justice, for instance by explaining what constitutes a fair allocation of environmental or climate burdens and benefits.

## Discussion

### Towards a Future Research agenda

Here, we discuss the implications of our key findings as the basis for a future research agenda capable of anticipatory governance of the environmental impacts of R&I, integrating the key concepts of environmental and climate ethics reviewed above.

#### Bridging the gap from the research ethics and integrity perspective

A key finding of this review is that research ethics and integrity literatures have little to say about the ethically significant impacts of research practices upon the environment. Many publications in business ethics, bioethics, and medical ethics that were reviewed in the first two stages of the review (title and abstract and full text screening) had little or no connection to environmental or climate justice/ethics and were therefore discarded based on the inclusion/exclusion criteria before we moved to the third stage of the review (data extraction). This could be due to our choice and/or interpretation of the inclusion/exclusion criteria. However, to anticipate the risk of being not sufficiently inclusive with our chosen criteria, we used a wide interpretation of the “substantial usage or analysis of the key topic areas” inclusion criterion: as long as the publication provided an explicit definition of the key topic area(s) discussed, or as long as it used the key topic area(s) as part of the methods methods/approaches or as part of findings/conclusions, it was included at the third stage (see Table S2 in the Supplementary Materials). This indicates that the literature on research ethics and integrity included in this review does not engage meaningfully with the environmental impacts of research, or the dimensions of ethics and justice which these may raise.

The history of research ethics may explain this result. Indeed, the very idea of including ethical considerations pertaining to the natural environment or our relationship to it is revolutionary when viewed in historical perspective. Since the Nuremberg Code was written in 1947, the focus of research ethics has been the protection of human participants in research projects, including through basic principles such as voluntary informed consent, protection of research participants, and the right to withdraw from an experiment. Subsequently, the focus of research ethics assessments such as those conducted by Research Ethics Boards (REC) and Institutional Review Boards (IRB) has been to protect human beings who participate in research. Research ethics guidelines were first developed in the field of biomedical research (WMA, 1964). They have later been extended to cover the interests of participants in social sciences research and the protection of animals (WMA, 2022). Nonetheless, this seems to imply a shortfall in the expertise necessary to consider the ethical dimensions of research and innovation pertaining to the environment.

In response, capacity building is required to enable research ethics and integrity assessors to evaluate these ethical dimensions of research and innovation. To do so, research ethics scholars and boards should more substantially engage with the insights available in environmental and climate ethics. They can also draw on the growing literature on the environmental and climate impact of research. A good example of this is the French Labos 1point5 collective, whose objective is to better understand and mitigate the impact of scientific research activities on the environment.^3^ They designed tools to measure and mitigate the environmental impact of research institutions (see e.g., Cluzel et al., 2020). Second, research ethics scholars and research ethics boards can focus upon the ethical and justice-related issues raised by the environmental impact of scientific research. The idea is that research ethicists and research ethics boards should acquire an expertise both on the environmental impact of research and on environmental and climate ethics. To do so, they could draw on some of the cross-cutting concepts discussed above (e.g., distributive, procedural, or epistemic justice), on the twenty basic principles for environmental research ethics put together by Curzer et al. (2013) (e.g., research that would harm an ecosystem greatly and would yield only small gains in knowledge should not be pursued), or on some of the recommendations from the CNRS Ethics Committee (2022) to integrate environmental issues into research practices (e.g., use the principles of equity or the do no harm principle to make the environment an integral part of research ethics).

Most research ethics and integrity approaches address aspects of good professional conduct and the virtues of science, research, and engineering. However, integrating the societal and ecological impacts of such work offers a different perspective on the role of environmental and climate ethics. “Micro-ethics” is concerned with the internal responsibility domain of science, research, and innovation and requires compliance with a set of guidelines or codes of conduct (Herkert, 2005). In contrast, “macro-ethics” understands ethical reflection and deliberation as an integral part of design work, which requires fulfilling external responsibility attributions. In recent years, a plethora of methods and tools for integrating applied ethical consideration into R&I work has been developed and provided, often framed under the umbrella of RRI and in the context of technology ethics (Gianni et al., 2018; Groot Kormelink, 2019). Examples of frameworks that reflect the normative implications of R&I include design thinking (Kerguenne et al., 2017), value-sensitive design/engineering (Friedman & Hendry, 2019), co-design (Heskett et al., 2017), embedded ethics (McLennan et al., 2022), ethical vision assessment (Grin & Grunwald, 2000), open innovation (Fritzsche et al., 2020), and ethics-by-design (Brey & Dainow, 2024; Mehlich & Woopen, 2025). These approaches seem more capable of translating insights from environmental ethics and climate ethics into research and innovation agendas than compliance approaches. However, this would also make significant demands upon the R&I actors’ expertise and capacities. A key to successfully integrating environmental and climate ethics into such R&I practices may therefore be the creation of interdisciplinary communication channels that allow for ongoing discourse within particular research and innovation cultures. The surprisingly weak link between environmental and climate ethics and RRI found in our review indicates that more work would need to be done to create such ongoing forms of collaboration between researchers and R&I actors.

#### Bridging the gap from the environmental and climate ethics perspective

Another key finding is that publications on environmental justice/ethics and climate justice/ethics had very little to say about ethical standards of research, with the notable exception of literature on geoengineering. The literatures on distributive justice, intergenerational justice, and so on, were nearly completely disconnected from the literature on research ethics and integrity. A way to address this gap would be for environmental ethicists and climate ethicists to engage more substantially with research ethics scholarship, and to pursue increased collaboration between scholars from these fields. This might be initiated and strengthened at two levels.

The first level is the development of teaching programmes that address both environmental and climate issues and R&I. To illustrate this, let us take the example of engineering programmes and how they could be equipped with an ecological perspective. Two pedagogical strategies put forward in the literature we have reviewed could contribute to a “greening” of the engineering curriculum. First, Goldman et al. (2014) discuss the socio-scientific issues (SSI) approach developed by Applebaum et al. (2010) and Tytler (2012). SSI relies on socio-moral discourse, argumentation, and debating, with the objective to involve learners in decision-making processes integrating the ethical dimensions of environmental challenges and engineering design choices. A second pedagogical strategy put forward by Seager et al. (2012) is sustainable engineering science (SES). SES is an integrative approach to science, education, and technology that adopts anticipatory approaches to unintended consequences resulting from technological innovation and that cultivates interactional expertise to facilitate cross-disciplinary exchanges that promote more sustainable engineering practices.

The second level where such collaboration could take place is in developing research ethics guidelines and frameworks that include the ethically significant impacts of research practices upon the environment. A first way to address this gap would be to update existing research ethics and integrity guidelines and frameworks to reflect environmental and climate ethics concerns, in the abovementioned macro-ethical sense. A second would be to develop new research ethics and integrity guidelines and frameworks, either by focusing on a specific environmental concern (e.g. climate change, biodiversity loss) or by focusing on the environmental implications of a specific research and innovation domain (e.g. AI, biotechnology).

### Limitations

This review has limitations that come from the search strings we designed. First, our review did not include publications focused on the environmental impact of some technologies and innovations, despite their apparent relevance. This was because such publications did not engage with the environmental ethics/justice and climate ethics/justice concepts, or with research ethics and research integrity concepts, and were therefore out of scope. The recent literature on the environmental impact of AI (e.g., Ahmad et al., 2021; Alloghani, 2024; Bolte & Van Wynsberghe, 2024; Van Wynsberghe, 2021) or of high-energy accelerators (e.g., Banerjee et al., 2023; Bloom & Boisvert, 2024; Breidenbach et al., 2023; Roser, 2022) are good illustrations of this: these publications cover the environmental impact of relevant domains of technological innovation, but they were not included because of their lack of engagement with the key topic areas discussed in this review. This is despite our wide interpretation of the “substantial usage or analysis of the key topic areas” inclusion criterion, as mentioned earlier.

A second limitation was the relative lack of literature included on indigenous perspectives, despite a specifically designed search string to capture this. Although we modified an existing search string that was used by the IPBES to find literature on indigenous and local knowledge holders in the context of biodiversity conservation, little of the literature we collected qualified under our inclusion criteria, again because of its lack of engagement with the key topic areas. Thus, literature on research ethics on indigenous lands and with Indigenous peoples as participants (e.g., Holmberg et al., 2023; O’Brien et al., 2024), was not identified by our searches despite its relevance for the environmental ethical and justice-related dimensions of research. There may be three reasons for this. First, some of this literature is not peer-reviewed. Second, this literature may not explicitly mention environmental or climate-related dimensions. Third, even if some publications did mention environmental concerns, they do not necessarily frame them in terms of environmental ethics/justice or climate ethics/justice.

Our review also involved limitations stemming from the databases we used. First, our results were ultimately limited to literature written in English. While a small number of results in French, German, and Spanish were initially included in the first step of the reviewing process, none were compatible with our inclusion criteria. We also attempted to find publications in specialised databases in French, German, and Spanish languages, but our complex search strings using proximity searches were not reproducible in these databases due to technical limitations. This limitation is somewhat mitigated by the fact that many authors from French, German, and Spanish-speaking countries also publish their research in English to contribute to international discussions in their research fields.^4^

A second and related limitation is that these databases tend to reflect the historical dominance of research institutions in Northern America and Europe and the research standards in place in these regions, contributing to the structural dominance of the Global North in academia (Schöpf, 2020). This is well illustrated by the publications that were included in our review. Although some studies were published by lead authors affiliated in Asia (8% of the selected literature), Africa (4%), Latin America (3%), and the Middle East (2%), the largest lead author affiliations were from the USA (43%), the EU (18%), and the UK (15%). Additionally, no European lead author affiliations came from outside the EU (except the UK and Switzerland), which may reflect the intra-European inequalities between the EU and its periphery. This could be in part due to the language specifications of the search, for example no search string included Arabic and hence there is no representation of North African or West Asian research institutes. Central and Southern Africa is also underrepresented, with only three articles. This dominance is also illustrated by the regions on which the empirical studies that were part of the reviewed literature were conducted. Although some case studies were conducted in Asia (8%), Africa (4%), Latin America (3%), and in the Middle East (2%), most were conducted in Northern America (19%) and Europe (10%).^5^ This underrepresentation reflects what Schöpf (2020) calls “academic dependency”, where many depend on finance and technology from dominant nations to secure academic legitimacy.

### The need for specific ethical governance of research and innovation

While this review aimed to find convergences among our key topic areas, there are good reasons not to over-generalise concepts beyond what the subject matter justifies. One of our most cited concepts illustrates this, namely that of geoengineering. This was one of the most discussed topics in the literature surveyed. It also managed to unify all key topic areas, as discussions of geoengineering often debate issues of intergenerational justice in the context of climate ethics, alongside issues of research governance as many geoengineering techniques are still in early stages of development. Further, ethical questions regarding impacts upon non-human nature are also discussed, although to a lesser extent.

However, the ethics of geoengineering raises unusually high risks compared with many standard R&I contexts. Consequently, the way this discourse has developed and its highly polarised character suggest it to be a poor model for integrating the environmental dimensions of R&I. Ethical issues raised by SRM and CDR techniques are sufficiently distinct that the label ‘geoengineering’ may be an obstacle to clear analysis of ethical and governance challenges (e.g., Heyward, 2013). SRM and CDR techniques remain highly controversial, and SRM research projects in particular have a history of being cancelled due to public opposition. This polarisation does not emerge from our review due to the study design, but it is widely acknowledged in the literature. Further, SRM is often compared with high-risk research areas including the development of biological, chemical and nuclear weapons, or human cloning (e.g. American Geophysical Union, 2024), which reflects unusually high degrees of ethical concern and elevated geopolitical stakes in the outcomes of research. These differences between techniques, as well as the high risks posed by SRM in particular, may explain the trend towards producing specific ethical guidelines for CDR as opposed to SRM, and the fact that the latter faces the strictest conditions on permissible research activities (e.g., European Commission, 2024; National Academies of Sciences, Engineering, and Medicine, 2021). As a result, we do not see the ethics of geoengineering as a promising model for integrating the environmental dimensions of R&I.

## Conclusion

To clarify the implications of ethical and justice perspectives pertaining to the environment and research ethics and integrity perspectives, this scoping review was limited to studies focused upon the environmental implications for innovation and technology development. While we anticipated that this would exclude much scientific literature on these perspectives, we did not anticipate the extent to which literature on research ethics and integrity would be excluded. This is especially surprising given the relative size of the wider literature on research ethics and research integrity. To give an illustration, a search in Scopus using our search string conducted on 7th February 2025 found 744 documents. Removing the requirement to mention either “climate” or “environment,” we found a much larger total of 3,121 documents. While a full screening of these databases would be necessary to precisely indicate the magnitude of literature that we excluded by introducing this requirement, this provides an indication of the size of research ethics and integrity literature which does not explicitly mention environmental impacts. This neglect of the environmental and climate dimensions of research and innovation was confirmed by the scoping review results highlighted above. Aside from the small set of studies utilising the RRI framework, we did not find concepts of research ethics or research integrity applied to contexts of environmental impact or concern.

Another main finding was the degree of fragmentation that exists in understandings of topics and concepts related to environmental ethics, environmental justice, and climate ethics and justice. Contrary to our initial expectations, seminal philosophical contributions on climate ethics were not widely cited in the literature surveyed, despite their significant impacts in wider literature on climate policy and justice. For instance, Shue’s article “Subsistence emissions and luxury emissions,” widely considered to be a foundational piece of scholarship on climate justice, was cited in merely two studies included in our review, despite having 403 citations in the Scopus database alone (Shue, 1993). As noted above, this indicates that philosophical conceptions of climate justice have little overlap with discussions of climate justice applied to research, innovation and technology. A less restricted review focused purely on the use of concepts is likely to present a very different picture of influential and cross-cutting concepts.

In contrast, the prominence of Schlosberg’s (2007) conception of environmental justice across the literature surveyed indicates that literature on this topic is less fragmentary, revealing a greater degree of shared understanding. The use of this in the fields of energy justice and digital justice bears this out. Nonetheless, even this finding should be viewed in the context of a larger fragmentation of understandings across the literature. While Schlosberg’s view is cited in 15 studies, a much larger set of literature reviewed did not refer to any shared understanding of environmental justice. This confirms our finding that the literature surveyed reveals considerable conceptual fragmentation, and work that seems to run parallel to similar research being done by scholars in different sub-fields or disciplines. A clear illustration is the topic of energy justice. As noted already, this literature most often cited the view of Schlosberg’s conception of environmental justice when defining itself, which seems to reveal a degree of conceptual rebranding. For instance, energy justice scholars McCauley and Heffron (2018, p. 1) claim that energy justice might unite work on climate and environmental justice in providing “a more comprehensive framework for analysing and ultimately promoting fairness and equity throughout the transition away from fossil fuels”. However, these seem to be reapplications of concepts of environmental justice (cf. Schlosberg, 2007), rather than new concepts in their own right.

Since responsibility stands out as one of the major cross-cutting concepts, it was surprising to find very few references to Jonas’s work on the imperative of responsibility (Jonas, 1984). Jonas was mentioned in the context of philosophy of technology (Sandler, 2020), and in discussion of how technology affected the human-nature relationship after the development of the nuclear bomb (Petit & Guillaume, 2018). Yet only one paper discussed Jonas’s imperative of responsibility in the context of environmental ethics, and this merely to indicate its limited relevance in the context of the Anthropocene (Cera, 2020).

Finally, among the environmental topics identified, the literature reveals an over-representation of studies focused upon climate change at the expense of other environmental issues. Most notable here may be research on biodiversity conservation, which is compared in importance and urgency with climate change (e.g., IPBES, 2019). Another over-represented topic was research on the ethics and governance of geoengineering, which was the second most discussed technology area in our review (after energy technologies). The prominence of this literature and its engagement with the RRI framework may reflect efforts by researchers to establish anticipatory governance of research prior to any physical experimentation due to the seriousness of concerns with ungoverned research. Nonetheless, we have suggested that the highly controversial character of this literature, and its unusually high risks, make it of limited relevance for broader work in research ethics that is inclusive of environmental concerns.

## Electronic supplementary material

Below is the link to the electronic supplementary material.

Supplementary Material 1

## Data Availability

Data included as electronic supplementary materials.

## Footnotes

1 This included the explicit definition of topics within a key topic area, such as definitions of biocentrism within the context of environmental ethics.

2 These are “Titanic risks” in the sense that they are related to the socio-economic as well as socio-geographic location of the victims. Just as in the case of the sinking of the Titanic, the poorest people in both the global north and the global south are the most exposed to climate impacts and most likely to lack the required economic and technological capital to adapt to climate change.

3 For more information on the activities of this collective, see the following website: https://labos1point5.org.

4 One example of this is the contribution by Petit and Guillaume (2018) that was included in the review, a book chapter from the volume *French Philosophy of Technology* that discusses the relation between political ecology, environmental ethics, and philosophy of technology in the context of French-speaking research.

5 The percentages do not add up to 100%, as they only track publications containing case studies, and many publications included in the review did not contain any case studies.
