# Supplementary material for: Convergences and Gaps between Environmental Ethics, Climate Ethics, and Research Ethics: A Scoping Review
Source: Sci Eng Ethics. 2026 Jan 12;32(1):11. doi: 10.1007/s11948-025-00575-8 (PMC12835071; doi:10.1007/s11948-025-00575-8)
Supplement: Supplementary file 1 — Supplementary Material 1 [file 11948_2025_575_MOESM1_ESM.docx]

**Supplementary Materials**

Table S1 – Search strings

| Database | Search string |
| --- | --- |
| Scopus | TITLE-ABS-KEY ( ( research W/3 ethic* ) OR ( research W/3 integrity ) OR ( climate W/3 ethic* ) OR ( environ* W/3 ethic* ) OR ( climate W/3 justice ) OR ( environ* W/3 justice ) ) AND KEY ( ( climate OR environ* ) AND ( technology OR innovation ) ) |
| Web of Science | TS=((research NEAR/3 ethic*) OR (research NEAR/3 integrity) OR (climate NEAR/3 ethic*) OR (environ* NEAR/3 ethic*) OR (climate NEAR/3 justice) OR (environ* NEAR/3 justice)) AND AK=((climate OR environ*) AND (technology OR innovation)) |
| Philosopher’s Index | SU ( ( research N3 ethic* ) OR ( research N3 integrity ) OR ( climate N3 ethic* ) OR ( environ* N3 ethic* ) OR ( climate N3 justice ) OR ( environ* N3 justice ) ) AND SU ( ( climate OR environ*) AND (technology OR innovation) ) |
| Scopus (Intercultural search string) | TITLE-ABS-KEY ( indigenous OR local ) AND ( pastoralis* OR herder OR fisher* OR hunt* OR forest* OR agriculture OR livelihood OR nomad OR land* OR river* OR sea OR ocean OR mountains ) AND ( knowledge OR philosophy ) AND ( values ) AND ( nature OR mother AND earth OR biodiversity OR ecosystem ) AND KEY ( ( climate OR environ* ) AND ( technology OR innovation ) ) |

Table S2 – Inclusion and exclusion criteria

| Inclusion Criteria | Exclusion Criteria |
| --- | --- |
| 1. Language: English, French, German, Spanish  2. Topic: Substantial usage or analysis of any of the following concepts or topic areas:   - Aspects of environmental ethics/justice - Aspects of climate ethics/justice - Aspects of sustainability ethics - Environmental risk - Research ethics/integrity (connected to environmental impact)   By ‘substantial usage or analysis’, we require articles to provide one or more of the following:   - - Explicit definitions of these concepts or topics, supported by reference to literature   - Usage of these concepts within analysis of the research conducted, either as part of methods/approaches or as part of findings/conclusions   3. Types of records: published scientific articles, review articles, book chapters, books | 1. Language: all other languages (incl. when abstract is in English but paper is in another language)  2. Wrong topic:   - - Where concepts or topics are peripheral to the analysis conducted, e.g. are included as potential further implications of the study that were not analysed within it;   - Where concepts or topics are not defined or referenced – corresponding to the conditions for exclusion for ‘generic usage’.   These conditions are the following:   - - Generic/vague usage of ‘ethics in research’ or ‘ethical issues in research’ or ‘research integrity’ without application to environmental sustainability or environmental impacts or synonyms   - Generic/vague usage of ‘ethics’ or ‘justice’ or ‘equity’ or ‘intergenerational justice’ or ‘social justice’ or ‘gender justice’ or ‘business ethics’ unconnected to environmental/climate impacts or environmental sustainability or synonyms   - Irrelevant/metaphorical usage of search terms with different meanings, e.g. ‘work environment’, ‘business climate’   - Use of ‘sustainability’ (e.g. corporate sustainability) with no mention of environmental sustainability or synonyms   3. Types of records:   - - Unpublished texts, e.g. grey literature, preprints   - Non-peer reviewed, e.g. letters, comments, editorials, book reviews, policy briefs, policy statements   - Duplicates   - Published in a predatory journal (peer review not verifiable), e.g. does not appear in the Directory of OA Journal (https://doaj.org/) or appears on the Predatory Journal's list (https://predatoryjournals.org/predatory-journals).   Note: When there are two versions of a paper, a proceeding/working paper version and a peer-reviewed journal-based publication, the latter is included. |

Figure S1 – Flow chart of selection process


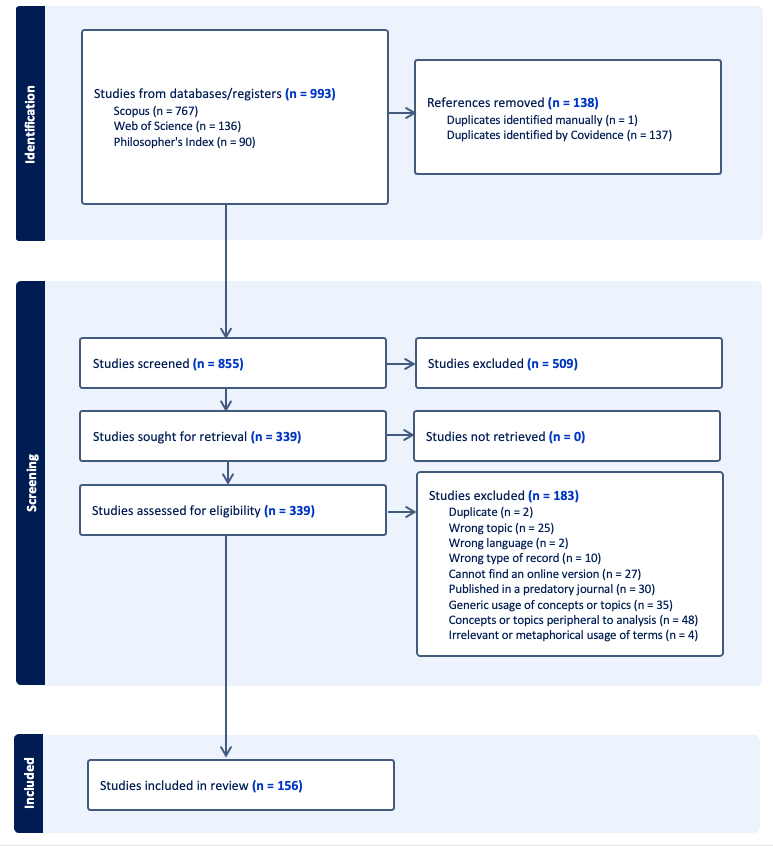


Table S3 – List of included studies

| Lead author name | Title | Year | Journal, chapter, or book title |
| --- | --- | --- | --- |
| Abel | States of environmental justice: Redistributive politics across the United States, 1993-2004 | 2015 | Review of Policy Research |
| Affolderbach | “Just” ecopreneurs: re-conceptualising green transitions and entrepreneurship | 2017 | Local Environment |
| Aftab | Environmental ethics, green innovation, and sustainable performance: Exploring the role of environmental leadership and environmental strategy | 2022 | Journal of Cleaner Production |
| Alario | Environmental Risks and Environmental Justice, Or How Titanic Risks Are Not So Titanic After All | 2010 | Sociological Inquiry |
| Allione | From ecodesign products guidelines to materials guidelines for a sustainable product. Qualitative and quantitative multicriteria environmental profile of a material | 2012 | Energy |
| Altman | The Role of Understanding, Trust, and Access in Public Engagement with Environmental Activities and Decision Making: A Qualitative Study with Water Quality Practitioners | 2023 | Environmental Management |
| Alvarez | Military, Race, and Urbanization: Lessons of Environmental Injustice from Las Vegas, Nevada | 2021 | Sociological Perspectives |
| Anthony | Building a Sustainable Future for Animal Agriculture: An Environmental Virtue Ethic of Care Approach within the Philosophy of Technology | 2012 | Journal of Agricultural and Environmental Ethics |
| Arancio | From inequalities to epistemic innovation: Insights from open science hardware projects in Latin America | 2023 | Environmental Science and Policy |
| Arias | Advancing the European energy transition based on environmental, economic and social justice | 2023 | Sustainable Production and Consumption |
| Arias Schreiber | Blue Justice and the co-production of hermeneutical resources for small-scale fisheries | 2022 | Marine Policy |
| Bailey | Toward environmental justice in transportation decision making with structured public involvement | 2012 | Transportation Research Record: Journal of the Transportation Research Board |
| Bassey | Technology, environmental sustainability and the ethics of anthropoholism | 2020 | Social Space |
| Batel | Energy Colonialism and the Role of the Global in Local Responses to New Energy Infrastructures in the UK: A Critical and Exploratory Empirical Analysis | 2016 | Antipode |
| Baum | Artificial Intelligence Needs Environmental Ethics | 2022 | Ethics, Policy & Environment |
| Behrsin | Controversies of justice, scale, and siting: The uneven discourse of renewability in Austrian waste-to-energy development | 2019 | Energy Research & Social Science |
| Bennett | Agricultural biotechnology: Economics, environment, ethics, and the future | 2013 | Annual Review of Environment and Resources |
| Bettini | Clouded skies: How digital technologies could reshape “Loss and Damage” from climate change | 2020 | WIREs Climate Change |
| Bhardwaj | Looking back, looking beyond: Revisiting the ethics of genome generation | 2006 | Journal of Biosciences |
| Biasetti | Insights toward an ethical assessment of CubeSat technologies | 2023 | Next Generation CubeSats and SmallSats: Enabling Technologies, Missions, and Markets |
| Biddle | Intellectual Property Rights and Global Climate Change: Toward Resolving an Apparent Dilemma | 2016 | Ethics, Policy & Environment |
| Biddle | Genetically engineered crops and responsible innovation | 2017 | Journal of Responsible Innovation |
| Bourban | Ethics, Energy Transition, and Ecological Citizenship | 2022 | Comprehensive Renewable Energy, Second Edition, Volume 9(15) |
| Briggle | Retail Sanity, Wholesale Madness: The Question Concerning Sustainability | 2009 | Philosophy in the Contemporary World |
| Brooks | Ethics of climate engineering: Don't forget technology has an ethical aspect too | 2022 | International Journal of Information Management 63 (2022) 102449 |
| Bush | Planning a just nature-based city: Listening for the voice of an urban river | 2023 | Environmental Science and Policy |
| Butterfield | The TERRA framework: Conceptualizing rural environmental health inequities through an environmental justice lens | 2009 | Advances in Nursing Science |
| Cera | The Anthropocene or the 'End' of the Imperative Responsibility | 2020 | Pensando - Revista de Filosofia |
| Chakladar | Paper use in research ethics applications and study conduct | 2011 | Clinical Medicine 2011, Vol 11, No 1: 44–7 |
| Chen | Utilize structural equation modeling (SEM) to explore the influence of corporate environmental ethics: The mediation effect of green human capital | 2013 | Quality & Quantity |
| Chen | Development of green business strategies through green dynamic capabilities and environmental regulation: Empirical evidence from the construction sector | 2024 | Journal of Cleaner Production |
| Chiles | Greenhouse Gas Emissions from Agriculture: Reconciling the Epistemological, Ethical, Political, and Practical Challenges | 2018 | Journal of Agricultural and Environmental Ethics |
| Cifuentes | Co-producing autonomy? Forest monitoring programs, territorial ontologies, and Indigenous politics in Amazonia | 2023 | Digital Geography and Society |
| Ciplet | Climate change and the transition to neoliberal environmental governance | 2017 | Global Environmental Change |
| Clarke | Future technologies, dystopic futures and the precautionary principle | 2005 | Ethics and Information Technology (2005) 7:121–126 |
| Clingerman | Thinking negatively about negative emissions technologies: the via negativa, carbon thinking, and climate ethics | 2022 | Journal of Environmental Studies and Sciences |
| Coeckelbergh | Environmental Virtue: Motivation, Skill, and (In)formation Technology | 2011 | Environmental Philosophy |
| Conti | Vulnerability and social justice as factors in emergent U.S. nanotechnology risk perceptions | 2011 | Risk Analysis |
| DeMarco | Rachel Carson's environmental ethic - a guide for global systems decision making | 2017 | Journal of Cleaner Production |
| Dzwonkowska | Environmental Ethics in Poland | 2017 | Journal of Agricultural and Environmental Ethics |
| Faran | Geoengineering: Neither economical, nor ethical—a risk– reward nexus analysis of carbon dioxide removal | 2018 | International Environmental Agreements |
| Farmer | Making morality: Sustainable architecture and the pragmatic imagination | 2010 | Building Research & Information |
| Feldman | Integrated water management and environmental justice - Public acceptability and fairness in adopting water innovations | 2011 | Water Science & Technology: Water Supply |
| Feron | Sustainability of rural electrification programs based on off-grid photovoltaic (PV) systems in Chile | 2016 | Energy, Sustainability and Societ |
| Flipse | Organizing a Collaborative Development of Technological Design Requirements Using a Constructive Dialogue on Value Profiles: A Case in Automated Vehicle Development | 2018 | Science and Engineering Ethics |
| Forsyth | When climate justice goes wrong: Maladaptation and deep co-production in transformative environmental science and policy | 2022 | Political Geography |
| Fraser | Biotechnology or organic? Extensive or intensive? Global or local? A critical review of potential pathways to resolve the global food crisis | 2015 | Trends in Food Science & Technology |
| Fulvi | Gambling on unknown unknowns: Risk ethics for a climate change technofix | 2024 | The Anthropocene Review |
| Gao | Solar adoption inequality in the U.S.: Trend, magnitude, and solar justice policies | 2022 | Energy Policy |
| Gerrie | Environmental ethics: Should we preserve the red herring and flounder? | 2003 | Journal of Agricultural and Environmental Ethics |
| Giampietro | Sustainable development: Scientific and ethical assessments | 1992 | Journal of Agricultural and Environmental Ethics |
| Gibson | Science Fiction Futures and (Re) visions of the Anthropocene | 2021 | The Oxford Handbook of Philosophy of Technology |
| Glynn | Ethical Issues in Environmental Decision Making and the Limitations of Cost/Benefit Analysis (CBA) | 1996 | Ethics and the Environment |
| Goldman | 'Human nature': Chemical engineering students' ideas about human relationships with the natural world | 2014 | European Journal of Engineering Education |
| Green | An analysis of some ethical argumentation about genetically modified food | 2024 | Argument & Computation |
| Gregg | Human genetic engineering: Biotic justice in the anthropocene? | 2017 | Encyclopedia of the Anthropocene |
| Guo | Do corporate environmental ethics influence firms’ green practice? The mediating role of green innovation and the moderating role of personal ties | 2020 | Journal of Cleaner Production |
| Hancock | In the lab: New ethical and supply chain protocols for battery and solar alternative energy laboratory research policy and practice | 2018 | Journal of Cleaner Production |
| Harris | Reflections on the value of ethics in relation to Earth observation | 2013 | International Journal of Remote Sensing |
| Hartley | Empowering Environmental Justice Decision Makers: Increasing Educational Resources for U.S. Environmental Protection Agency's Mapping Tools | 2021 | Environmental Justice |
| Heide | Embedded but overlooked values: Ethical aspects of absolute environmental sustainability assessments | 2024 | Journal of Industrial Ecology |
| Hess | What is a clean bus? Object conflicts in the greening of urban transit | 2007 | Sustainability: Science, Practice and Policy |
| Hilser | Localized governance of carbon dioxide removal in small island developing states | 2024 | Environmental Development |
| Hofbauer | Systemic risks and solar climate engineering research. Integrating technology ethics into the governance of systemic risks | 2023 | Journal of Risk Research |
| Hoffman | Environmental justice along product life cycles: importance, renewable energy examples and policy complexities | 2017 | Local Environment: The International Journal of Justice and Sustainability |
| Hope | The impact of religious faith on attitudes to environmental issues and Carbon Capture and Storage (CCS) technologies: A mixed methods study | 2014 | Technology in Society |
| Hornborg | Colonialism in the anthropocene: The political ecology of the money-energytechnology complex | 2019 | Journal of Human Rights and the Environment |
| Huang | Shaping environmental justice: Applying science; technology and society boundary work | 2012 | Electronic Green Journal |
| Hughes | Greenwashing in Palestine/Israel: Settler colonialism and environmental injustice in the age of climate catastrophe | 2023 | EPE: Nature and Space |
| Jamison | Climate change knowledge and social movement theory | 2010 | WIREs climate change |
| Johnston | Environmental justice and dissent forpostcolonial urban sustainability transitions | 2023 | International Journal of Urban and Regional Research |
| Karki | Potentials and barriers to land-based mitigation technologies and practices (LMTs)—a review | 2023 | Environmental Research Letters |
| Karlsson | Individual guilt or collective progressive action? Challenging the strategic potential of environmental citizenship theory | 2012 | Environmental Values |
| Kaul | Public value mapping to assess and guide governmental investments in energy and environmental justice: Studying the United States Department of Energy | 2023 | Renewable and Sustainable Energy Reviews |
| Khayat | From Climate Injustice to Resilience: What Is the Role of Social and Technological Innovation? | 2023 | Environmental Justice |
| Kim | Green infrastructure and energy justice in health adaptation: leveraging climate policy innovation and vulnerability-readiness nexus | 2022 | Journal of Environmental Policy & Planning |
| King | Playing with Boundaries: Critical Reflections on Strategies for an Environmental Culture and the Promise of Civic Environmentalism | 2006 | Ethics, Policy & Environment |
| Kinol | Climate justice in higher education: a proposed paradigm shift towards a transformative role for colleges and universities | 2023 | Climatic Change |
| Kurochkin | Energy Policy Advancement: Climate Change Mitigation and International Environmental Justice | 2022 | (full book) |
| LaBelle | In pursuit of energy justice | 2017 | Energy Policy |
| Larrabee | Climate change and conflicting future visions | 2018 | Zygon |
| Larrère | Two philosophies of the environmental crisis | 2013 | The Structural Links between Ecology, Evolution and Ethics: The Virtuous Epistemic Circle |
| Lawrence | Ignorance as strategy: ‘Shadow places’ and the social impacts of the ranger uranium mine | 2022 | Environmental Impact Assessment Review |
| Levenda | Renewable energy for whom? A global systematic review of the environmental justice implications of renewable energy technologies | 2021 | Energy Research and Social Science |
| Lloro-Bidart | Intersectional feminism for the environmental studies and sciences: looking inward and outward | 2018 | Journal of Environmental Studies and Sciences |
| Low | The practice of responsible research and innovation in “climate engineering” | 2020 | WIREs Climate Change |
| Lynch | Second-Guessing Scientists and Engineers: Post Hoc Criticism and the Reform of Practice in Green Chemistry and Engineering | 2015 | Science and Engineering Ethics |
| Mabon | Meeting the targets or re-imagining society? An empirical study into the ethical landscape of carbon dioxide capture and storage in Scotland | 2015 | Environmental Values |
| Macdonald | Indigenous-led responsible innovation: lessons from co-developed protocols to guide the use of drones to monitor a biocultural landscape in Kakadu National Park, Australia | 2021 | Journal of Responsible Innovation |
| Mahlanza | Water, Rights and Poverty: an Environmental Justice Approach to Analysing Water Management Devices in Cape Town | 2016 | Urban Forum |
| Mahoney | The Virtue of Burden and Limits of 'Gelassenheit': The Complex Case for Heideggerian Environmental Ethics | 2016 | Environmental Philosophy |
| Mancilla | Climate justice and territory | 2023 | WIREs Climate Change |
| McAllister | Women, E-waste, and technological solutions to climate change | 2014 | Health and Human Rights |
| McCauley | Just transition: Integrating climate, energy and environmental justice | 2018 | Energy Policy |
| McGuire | Increased exposure to environmental hazards: An opportunity for science, technology, engineering, and math education | 2018 | Environmental Justice |
| Miller | The Moral Philosophy of Automobiles | 2012 | Journal of Agricultural and Environmental Ethics |
| Mintz-Woo | The NET effect: Negative emissions technologies and the need-efficiency trade-off | 2023 | Global Sustainability |
| Muraca | Viable and convivial technologies: Considerations on Climate Engineering from a degrowth perspective | 2018 | Journal of Cleaner Production |
| Nawaz 2024 | Towards just, responsible, and socially viable carbon removal: lessons from offshore DACCS research for early-stage carbon removal projects | 2023 | Environmental Science and Policy |
| Nost 2022 | Earth for AI: A Political Ecology of Data-Driven Climate Initiatives | 2022 | Geoforum |
| Ogiemwonyi | Environmental ethics and green practices in the manufacturing sector: The role of green innovation and environmental policy | 2024 | Natural Resources Forum |
| Okada | Social responsibility for the use of genes, genomes and biotechnology in biotechnology companies: A commentary from the bioethical viewpoint | 2008 | Journal of Commercial Biotechnology |
| Okereke | Climate justice and the international regime | 2010 | WIREs Climate Change |
| Orozco-Meléndez | A role for grassroots innovation toward agroecological transitions in the Global South? Evidence from Mexico | 2022 | Ecological Economics |
| Ottinger | Environmentally just technology | 2011 | Environmental Justice |
| Ottinger | Environmentally just transformations of expert cultures: Toward the theory and practice of a renewed science and engineering | 2012 | Environmental Justice |
| Pamplany | The Ethics of Geoengineering: A Literature Review | 2020 | Science and Engineering Ethics |
| Parris-Piper | Automating violence? The anti-politics of ‘smart technology’ in biodiversity conservation | 2023 | Biological Conservation |
| Perovich | Pokémon Go, pH, and projectors: Applying transformation design and participatory action research to an environmental justice collaboration in Chelsea, MA | 2018 | Cogent Arts & Humanities |
| Petit | We Have Never Been Wild: Towards an Ecology of the Technical Milieu | 2018 | French Philosophy of Technology |
| Popke | A social justice framing of climate change discourse and policy: Adaptation, resilience and vulnerability in a Jamaican agricultural landscape | 2014 | Geoforum |
| Powell | Technologies of existence: The indigenous environmental justice movement | 2006 | Development |
| Purvis | A framework for a responsible circular economy | 2023 | Journal of Cleaner Production |
| Rehmann-Sutter | Nature in the laboratory - nature as a laboratory. Considerations about the ethics of release experiments | 1993 | Experientia |
| Roe | Understanding the role of wrongdoing in technological disasters: Utilizing ecofeminist philosophy to examine commemoration | 2021 | Studies in History and Philosophy of Science |
| Ross | Intersections of disadvantaged communities and renewable energy potential: Data set and analysis to inform equitable investment prioritization in the United States | 2022 | Renewable Energy Focus |
| Rossi | Poor online information on European marine protected areas impairs public participation under the Aarhus Convention | 2024 | Marine Policy |
| Rui | Stakeholder pressure, corporate environmental ethics and green innovation | 2020 | Asian Journal of Technology Innovation |
| Sackey | One-Size-Fits-None: A Heuristic for Proactive Value Sensitive Environmental Design | 2020 | Technical Communication Quarterly |
| Samet | Urban Air Pollution and Health Inequities: A workshop report | 2001 | Environmental Health Perspectives |
| Sandler | The ethics of genetic engineering and gene drives in conservation | 2019 | Conservation Biology |
| Saville | Balancing environmental remediation, environmental justice, and health disparities: The case of Lake Apopka, Florida | 2019 | Case Studies in the Environment |
| Scammell | Tools for addressing cumulative impacts on human health and the environment | 2014 | Environmental Justice |
| Scott | Something in the Air: Civic science and contentious environmental politics in post-apartheid South Africa | 2009 | Geoforum |
| Seager | Sustainable Engineering Science for Resolving Wicked Problems | 2012 | Journal of Agriculture and Environmental Ethics |
| Segovia | Transforming Mindsets Through Education for Sustainable Development | 2010 | International Encyclopedia of Education |
| Shaw | The Bill and Melinda Gates Foundation and the necro-populationism of ‘climate-smart’ agriculture | 2019 | Gender, Place & Culture. A Journal of Feminist Geography |
| Shrader-Frechette | Environmental Ethics | 2003 | The Oxford Handbook of Practical Ethics |
| Shulman | Empowering environmentally-burdened communities in the US: A primer on the emerging role for information technology | 2006 | Local Environment. The International Journal of Justice and Sustainability |
| Simsek | Investigation of Environmental Topics in the Science and Technology Curriculum and Textbooks in Terms of Environmental Ethics and Aesthetics | 2011 | Educational Sciences: Theory & Practic |
| Skillington | Embracing sustainable development: The role of business in the communication and application of environmental ethics | 1996 | Sustainable Development |
| Snyder | Beyond the social cost of carbon: Negative emission technologies as a means for biophysically setting the price of carbon | 2020 | Ambio |
| Stephens | Financial innovation for climate justice: central banks and transformative ‘creative disruption’ | 2024 | Climate and Development |
| Stephens | The dangers of masculine technological optimism: Why feminist, antiracist values are essential for social justice, economic justice, and climate justice | 2024 | Environmental Values |
| Stevenson | Governing Climate Technologies: Is There Room for Democracy? | 2013 | Environmental Values |
| Stone | Towards a Darker Future? Designing Environmental Values into the Next Generation of Streetlights | 2021 | Technology and the City |
| Stretesky | The Role of Institutional Trust in Industry, Government, and Regulators in Shaping Perceptions of Risk Associated with Hydraulic Fracturing in the United Kingdom | 2023 | Sociological Perspectives |
| Sun | A new method for dividing the scopes and priorities of air pollution control based on environmental justice | 2021 | Environmental Science and Pollution Research |
| Symons | Ecomodernist citizenship: rethinking political obligations in a climate-changed world | 2018 | Citizenship Studies |
| Tanveer | Ethics, pandemic and environment; looking at the future of low middle income countries | 2020 | International Journal for Equity in Health |
| Tomblin | The ecological restoration movement: Diverse cultures of practice and place | 2009 | Organization & Environment |
| Tsekos | Ethics, science and environment: The need for a new environmental worldview | 2009 | International Journal of Environmental Studies |
| Tucker | Can Science and Religion respond to climate change? | 2015 | Why Do We Disagree on Climate Change? |
| Vaishnav | Implications of Green Technologies for Environmental Justice | 2023 | Annual Review of Environment and Resources |
| vanWynsberghe | The Dawning of the Ethics of Environmental Robots | 2018 | Science and Engineering Ethics |
| Verharen | African Environmental Ethics: Keys to Sustainable Development Through Agroecological Villages | 2021 | Journal of Agricultural and Environmental Ethics |
| Werse | The quest to cultivate an ecocritical awareness in educational technology scholarship: A question of disciplinary focus in the age of environmental crisis | 2023 | British Journal of Educational Technology |
| Wiseman | Hope and Courage in a Harsh Climate: From Denial and Despair to Resilience and Transformation | 2022 | The Palgrave Handbook of Climate Resilient Societies |
| Wong | Making power explicit in sustainable water innovation: Re-linking subjectivity, institution and structure through environmental citizenship | 2009 | Environmental Politics |
| Wyeth | Integrating Citizen Science into the Work of United States Environmental Agencies | 2023 | Citizen Science: Theory and Practice |
| Xia | Opportunities and challenges of interdisciplinarity in river water environmental ethics and integrated river basin management | 2024 | River |
| Xie | Advancing eco-excellence: Integrating stakeholders’ pressures, environmental awareness, and ethics for green innovation and performance | 2024 | Journal of Environmental Management |
| Yearley | The ethical landscape: Identifying the right way to think about the ethical and societal aspects of synthetic biology research and products | 2009 | Journal of the Royal Society Interface |
| Yen | The impact of ecological innovation and corporate social responsibilities on the sustainable development: Moderating role of environmental ethics | 2023 | Economic Research-Ekonomska Istraživanja |
| Ying | A fair trade? Expert perceptions of equity, innovation, and public awareness in China’s future Emissions Trading Scheme | 2021 | Climatic Change |
| Ziegler | Embedding circularity: Theorizing the social economy, its potential, and its challenges | 2023 | Ecological Economics |
